# Le syndrome des brides amniotiques et ses difficultés diagnostiques et de prise en charge au Burkina Faso

**DOI:** 10.11604/pamj.2015.20.208.6129

**Published:** 2015-03-06

**Authors:** Kisito Nagalo, Roger Badiel, Fla Kouéta, François Housséini Tall, Diarra Yé

**Affiliations:** 1 Service de Pédiatrie de la Clinique El Fateh-Suka, Ouagadougou, Burkina Faso; 2 UFR/SDS Université de Ouagadougou, Burkina Faso; 3 Société Burkinabè de Pédiatrie, Ouagadougou, Burkina Faso

**Keywords:** Maladie des brides amniotiques, Limb Body Wall Complex, embryo-fœtopathies, conseil génétique, Amniotic Bands Syndrome, Limb Body Wall Complex, embryofoetopathies, genetic counseling

## Abstract

Le syndrome des brides amniotiques est une embryo-foetopathie rare, d’étiopathogénie encore inconnue, caractérisé par des malformations crânio-faciales, thoraco-abdominales, des membres et des extrémités. Afin de discuter des difficultés diagnostiques et thérapeutiques du syndrome des brides amniotiques, nous rapportons cinq cas de ce syndrome. Ces cas représentaient autant de phénotypes de la maladie mais avec quelques singularités. Les deux premiers étaient des cas de maladie des brides amniotiques caractérisés l'un par une amputation d'un membre inférieur associée à des lésions cutanées et à une surdité, l'autre par des strictions avec amputation des doigts associées à une fente labio-palatine, une cataracte congénitale et un strabisme. Les trois autres cas correspondaient à des formes létales du Limb Body Wall Complex dont deux avec attache placento-crânienne et un avec attache placento-abdominale. Le renforcement du diagnostic anténatal, l'instauration du conseil génétique et la mise en place d'un registre national des malformations devraient permettre d'améliorer la prise en charge des cas du syndrome des brides amniotiques.

## Introduction

Le syndrome de brides amniotiques (SBA) est une malformation congénitale relativement rare. Les malformations qui intéressent principalement les extrémités mais aussi le crâne, la face et l'axe thoraco-abdominal peuvent se rencontrer sous deux formes: la maladie des brides amniotiques (MBA) et le syndrome Limb Body Wall Complex (LBWC) [1]. La pathogénie du SBA n'est toujours pas clairement établie mais deux théories sont proposées pour l'expliquer: d'une part, la théorie exogène par rupture de l'amnios conduisant à des bandes fibreuses qui vont stranguler le corps du fœtus et, d'autre part, la théorie endogène qui privilégie l'atteinte vasculaire [2, 3]. Une grande variété de déformations cliniques est rencontrée, allant des rétrécissements annulaires simples et des défauts mineurs numériques à des malformations majeures crânio-faciales et viscérales [1, 4]. Selon le milieu, le SBA peut poser aux praticiens des difficultés de prise en charge qui sont fonction non seulement du niveau du plateau technique mais également de considérations morales et religieuses. Nous rapportons cinq cas du SBA afin de rappeler la diversité des formes cliniques et présenter les difficultés diagnostiques et thérapeutiques dans un contexte de ressources limitées au Burkina Faso.

## Patient et observation

### Observation N° 1

**Figure 1:**
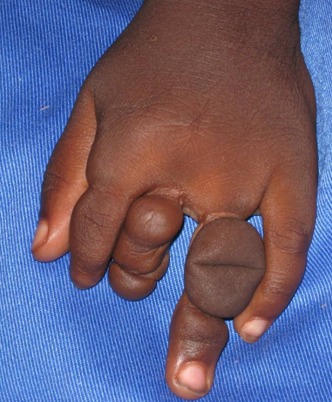
Maladie des brides amniotiques: sillons de striction des 2e, 3e et 4e doigts de la main gauche. On devine l'amputation de la dernière phalange des 2e et 3e doigts

Cet enfant de sexe masculin, âgé de 3 ans, était vu en consultation pour la prise en charge de sa fente labio-palatine. Son père (33 ans, informaticien) et sa mère (26 ans, commerçante) n’étaient pas consanguins. L'enfant était l'aîné d'une fratrie de 2 enfants, son petit frère était en bon état apparent de santé; il n'y avait pas de malformation dans la famille. La grossesse et l'accouchement étaient normaux. L'examen clinique montrait une fente labio-palatine unique gauche, une cataracte congénitale et un strabisme de l’œil gauche. Sa main gauche montrait une amputation des dernières phalanges des 2^ème^ et 3^ème^doigts avec des sillons de striction intéressant les 2^ème^, 3^ème^ et 4^ème^ doigts (Figure 1). Ces signes ont permis de poser le diagnostic de MBA. Une staphylorraphie réparatrice de sa fente a été faite, de même qu'une plastie en Z des doigts. L’évolution était favorable.

### Observation N° 2

**Figure 2:**
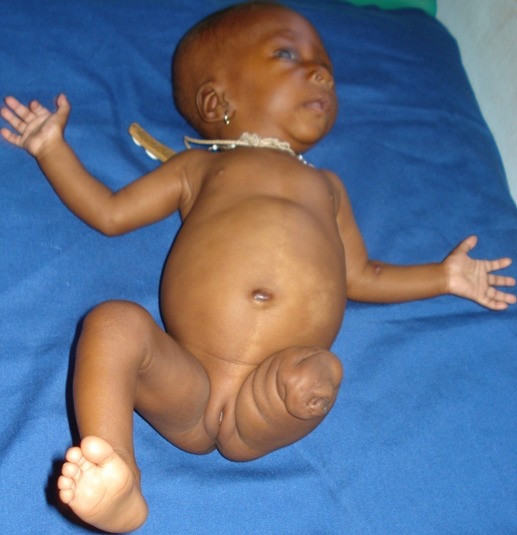
Maladie des brides amniotiques: amputation totale de la jambe et du tiers inférieur de la cuisse. Le moignon laisse voir l'orifice d'où a été extrait le fragment osseux. On note les lésions cutanées hypochromiques de la ligne médiane et para-abdominales gauches

Ce nourrisson de sexe féminin, était reçu en consultation à l’âge de 3 mois pour malformation congénitale du membre inférieur gauche. Sa mère, âgée de 21 ans, G1P0, n'avait pas d'antécédent médical particulier. L'anamnèse infectieuse au cours de la grossesse était négative. Une échographie obstétricale réalisée à 32 SA notait un oligoamnios mais ne décelait pas d'anomalie morphologique du fœtus. L'accouchement était par voie basse à l'issue d'une grossesse de 33 SA. Le nouveau-né était un prématuré modéré et pesait 1700 g à la naissance. L'examen clinique notait une dysmélie du membre inferieur gauche caractérisée par une amputation totale de la jambe et du tiers inferieur de la cuisse. La palpation de l'extrémité du moignon a permis de retirer un fragment osseux d'environ 1 cm x 2 mm; les autres membres et extrémités étaient normaux. Des lésions cutanées hypochromiques étaient observées sur un abdomen qui était proéminent avec une petite hernie ombilicale non étranglée à collet très étroit. Par ailleurs la chevelure était peu fournie et fine et on notait un léger strabisme divergent (Figure 2). L'examen neurologique et celui des autres appareils étaient normaux apparemment. L’échographie-doppler cardiaque montrait un shunt gauche-droit à travers un foramen ovale perméable et une insuffisance tricuspide de grade I-II tandis que l’échographie abdomino-pelvienne à la recherche d'autres malformations était normale. Devant tous ces signes cliniques, le diagnostic de MBA était posé. Le suivi de l'enfant révélait une surdité de transmission. Une évacuation sanitaire humanitaire aux Etats-Unis a permis de poser une prothèse de jambe et des implants auditifs. L’évolution était bonne à 3 ans.

### Observation N° 3

**Figure 3:**
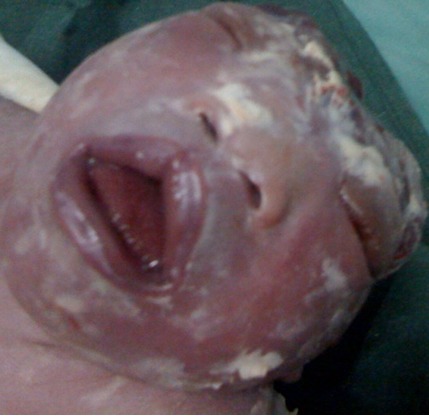
Limb Body Wall Complex avec attache placento-crânienne: anencéphalie, anomalies de la face (visage aplati, absence d'ensellure nasale, hypertélorisme, fente labiale droite incomplète), macroglossie

Il s'agissait d'un nouveau-né de sexe masculin né par césarienne à 40 SA. Ses parents n’étaient pas consanguins. La mère, âgée de 29 ans était G1P0 et n'avait pas d'antécédents particuliers. Au cours de la grossesse qui était bien suivie, deux échographies réalisées à 30 et 32 SA montraient une anencéphalie. L'interruption de la grossesse a été proposée au couple qui l'a refusée pour des raisons d'ordre morale et religieuse. A la naissance, le nouveau-né qui pesait 2500 g était en état de mort apparent. L'examen montrait une microcéphalie avec absence de la voûte crânienne révélant l'anencéphalie. Une fente labiale droite incomplète, une macroglossie, des oreilles malformées et bas implantées, une dysmorphie faciale avec un visage aplati d'avant en arrière et un hypertélorisme (Figure 3) ainsi qu'une ambiguïté sexuelle étaient aussi notés. Le reste de l'examen était normal. Le nouveau-né est décédé quelques heures après sa naissance. L'ensemble de ces signes cliniques a permis de poser de façon rétrospective le diagnostic de LBWC avec attache placento-crânienne.

### Observation N° 4

**Figure 4:**
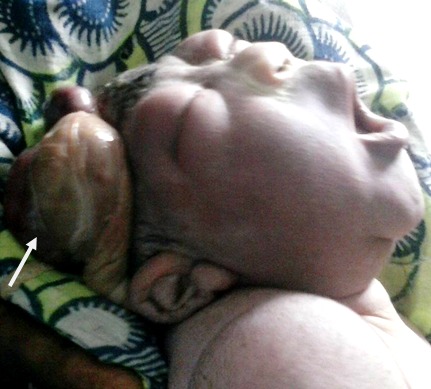
Limb Body Wall Complex avec attache placento-crânienne: reste de l'amnios (flèche) adhérant aux structures crânio-cérébrales.

Cette femme âgée de 30 ans, G5P4 était reçue pour des douleurs abdomino-pelviennes sur une grossesse à terme. Aucune échographie obstétricale n'a été réalisée durant la grossesse. L'accouchement était normal et le nouveau-né était dans un état de mort apparent avec un poids de 3350 g, un périmètre crânien d'environ 36 cm et une taille de 50 cm; l'examen physique montrait un défect du crâne avec l'amnios qui adhérait au cuir chevelu et à une partie des structures cérébrales, une encéphalocèle latérale droite, un œdème palpébral bilatéral (Figure 4), une anomalie de l'ensellure nasale, l'absence de philtrum. On notait aussi une ambiguïté sexuelle; le reste de l'examen semblait normal. Le nouveau-né est décédé environ 4 heures après sa naissance. Ce syndrome polymalformatif a permis de poser le diagnostic de LBWC dans sa forme avec attache placento-crânienne.

### Observation N° 5

**Figure 5:**
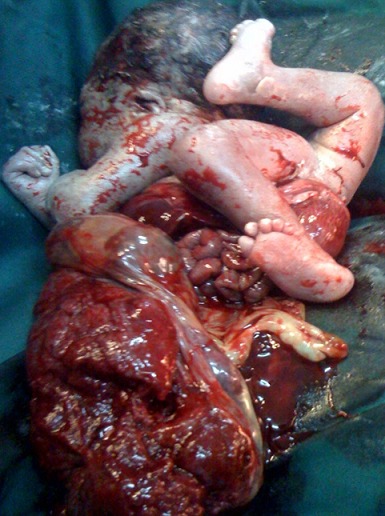
LBWC avec attache placento-abdominale: abdominoschisis avec éviscération des organes intra-abdominaux et placenta adhérent, hyperlordose avec torsion du rachis, position vicieuse des membres inferieurs, pied gauche valgus abductus

Ce nouveau-né est né prématuré à 32 SA. Ses parents n'avaient pas de liens de consanguinité. Sa mère, âgée de 32 ans, G7P1, avec un antécédent de mort-né, était admise en maternité pour menace d'accouchement prématuré sur une grossesse qui avait bénéficié d'un cerclage à 13 SA. Elle était obèse et hypertendue connue sous traitement. L’échographie notait un oligo-amnios sévère, une hydronéphrose rénale gauche avec une méga-vessie mais elle ne décelait pas d'autres anomalies morphologiques du fœtus. L'uroculture avait identifié *Proteus mirabilis*. Au 4^ème^ jour d'hospitalisation, malgré le traitement, il y avait une perte continue des eaux, des contractions utérines de plus en plus rapprochées avec une forte angoisse maternelle. Une césarienne a permis d'extraire en bloc un fœtus avec son placenta. Le nouveau-né présentait un abdominoschisis avec éviscération des principaux viscères intra-abdominaux qui étaient reliés au placenta, une hyperlordose avec torsion du rachis lombaire et une malrotation du membre inférieur droit l'amenant jusque dans le dos, un pied gauche valgus abductus (Figure 5). Le nouveau-né est décédé 30 minutes environ après la naissance. Le diagnostic d'une forme avec attache placento-abdominale du LBWC était posé de façon rétrospective.

## Discussion

L'incidence du SBA est difficile à préciser en raison de la grande diversité de ses présentations cliniques. Dans les pays développés certains auteurs estiment son incidence entre 0,11/10000 [5] et 1600 naissances [1]. En Afrique, son incidence n'est pas connue, les cas rapportés proviennent de données hospitalières [6–8]; beaucoup de cas ne sont certainement pas rapportés. Au Burkina Faso, les cas que nous rapportons sont les premiers à notre connaissance alors que les malformations congénitales sont relativement fréquentes en pratique quotidienne. Il convient non seulement de renforcer les capacités diagnostiques des praticiens par la formation et l'amélioration du plateau technique mais aussi d'améliorer la notification des cas en mettant en place un registre national des malformations congénitales. Le diagnostic du SBA doit être anténatal, les anomalies crânio-faciales et thoraco-abdominales pouvant être visualisées dès la première échographie à 10-12 SA [9–11]. Dans notre série, le diagnostic dans les observations N° 3 et 5 avait été porté tardivement tandis que la malformation était même passée inaperçue dans l'observation N° 2 malgré l’échographie obstétricale et elle n'a été découverte que dans la petite enfance dans l'observation N° 1. Dans les pays pauvres d'Afrique sub-saharienne comme le Burkina Faso, l’échographie obstétricale n'est pas toujours accessible à toutes les femmes enceintes comme dans l'observation N° 4. Dans ces conditions, il difficile de faire un dépistage précoce des anomalies fœtales. Même quand l’échographie est disponible, ce sont des facteurs techniques (faible performance des appareils) et/ou humains (inexpérience de l’échographiste et sa méconnaissance des pathologies malformatives) qui peuvent constituer des limites au diagnostic anténatal [12]. A la naissance, le diagnostic du SBA est purement clinique. Selon la classification de Levy et al. [1], les observations N° 1 et 2 correspondaient à la MBA qui est de meilleur pronostic, caractérisée essentiellement par des anomalies des membres et des extrémités qui peuvent être associées à d'autres malformations. Parmi ces malformations associées au SBA, la fente labio-palatine décrite dans l'observation N° 1 est déjà rapportée [13] mais les anomalies oculaires paraissent inhabituelles. De même, les lésions cutanéo-phanériennes, oculaires et ORL chez un nourrisson de sexe féminin (observation N° 2) semblent exceptionnelles et elles pouvaient évoquer une dysplasie congénitale méso-ectodermique du type syndrome de Goltz mais faute de moyens d'investigation, ce diagnostic n'a pu être confirmé. Dans le LBWC, les malformations sont sévères, létales et intéressent d'une part le crâne et la face à type d'anencéphalies, d'exencéphalies, d'encéphalocèle, de fentes faciales plus ou moins complexes et d'autre part le thorax et l'abdomen à type de thoraco-abdominoschisis. Les observations N° 3, 4 et 5 peuvent être classées dans cette forme avec les deux phénotypes décrits par Russo et al. [14], à savoir la forme avec attache placento-crânienne (observations N° 3 et 4) et celle avec attache placento-abdominale (observation N° 5). Le pronostic du SBA est fonction de la sévérité des malformations. Dans les formes peu sévères, un traitement palliatif chirurgical [6, 11] ou prothétique (observations N° 1 et 2) peut permettre au patient d'avoir une qualité de vie optimale. Par contre, le LBWC est au-dessus de toute ressource thérapeutique, le décès est inéluctable soit in utero soit peu après la naissance [8, 15], ce que confirme les observations N° 3, 4 et 5. L'interruption de la grossesse semble donc raisonnable dans ces cas [11, 15] mais en Afrique, elle est encore difficilement acceptée par les patients pour des raisons socioculturelles et religieuses, ce qui a expliqué le refus du couple dans l'observation N° 3.

## Conclusion

Le syndrome des brides amniotiques est un ensemble malformatif ubiquitaire dont les présentations cliniques sont aussi variées qu'inhabituelles. Afin d'améliorer le diagnostic dans les pays en développement, il est nécessaire de créer des centres référence de génétique humaine et de fœtopathologie, des registres nationaux des malformations congénitales, de disposer d’équipements performants et de spécialistes (échographistes, obstétriciens, pédiatres) très bien avisés sur les malformations de l'enfant. La prise en charge thérapeutique qui est multidisciplinaire reste aussi un challenge dans nos pays à ressources limitées.
